# Roles of Nuclear Pores and Nucleo-cytoplasmic Trafficking in Plant Stress Responses

**DOI:** 10.3389/fpls.2017.00574

**Published:** 2017-04-12

**Authors:** Yu Yang, Wei Wang, Zhaoqing Chu, Jian-Kang Zhu, Huiming Zhang

**Affiliations:** ^1^Shanghai Center for Plant Stress Biology, Shanghai Institutes for Biological Sciences, Chinese Academy of SciencesShanghai, China; ^2^Shanghai Key Laboratory of Plant Functional Genomics and Resources, Shanghai Chenshan Botanical GardenShanghai, China; ^3^Shanghai Chenshan Plant Science Research Center, Chinese Academy of SciencesShanghai, China; ^4^Department of Horticulture and Landscape Architecture, Purdue University, West LafayetteIN, USA

**Keywords:** nuclear pore complex, nucleoporin, abiotic stress, biotic stress, nucleo-cytoplasmic transport

## Abstract

The nuclear pore complex (NPC) is a large protein complex that controls the exchange of components between the nucleus and the cytoplasm. In plants, the NPC family components play critical roles not only in essential growth and developmental processes, but also in plant responses to various environmental stress conditions. The involvement of NPC components in plant stress responses is mainly attributed to different mechanisms including control of mRNA/protein nucleo-cytoplasmic trafficking and transcriptional gene regulation. This mini review summarizes current knowledge of the NPC-mediated plant stress responses and provides an overview of the underlying molecular mechanisms.

## Introduction

**FIGURE 1:**
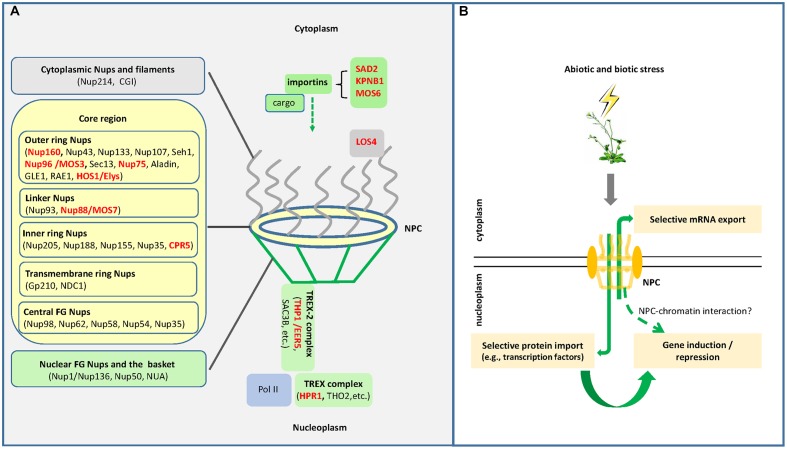
**(A)** Schematic representation of the plant nuclear pore complex (NPC) and its associated factors. The NPC consists of the outer cytoplasm region, the symmetrical core region and the inner nucleoplasm region. The symmetrical core region is composed of the outer ring nucleoporins (Nups), the linker Nups, the inner ring Nups, the transmembrane ring Nups, and the central FG Nups (nucleoporins rich in phenylalanine–glycine repeats) (modified from Tamura and Hara-Nishimura, 2013). The nucleoplasm region is associated with the TREX-2 (transcription-coupled export 2) complex that, together with the TREX complex, couples Pol II transcription with mRNA export. Also shown are some importins and the LOS4 protein, which mediate protein/RNA nucleoplasmic trafficking as well as plant stress responses. Red names indicate proteins involved in plant stress responses. **(B)** The NPC functions in plant stress responses through diverse mechanisms. In plants under stress conditions, the NPC may selectively export stress-responsive mRNAs into the cytoplasm for protein synthesis; the NPC may also selectively import certain proteins such as stress-responsive transcription factors for transcriptional regulation; the NPC may also directly regulate gene expression at the transcriptional level through NPC-chromatin interactions.

The nuclear pore complex (NPC) is the gateway of macromolecular trafficking between the nucleus and the cytoplasm (Xu and Meier, 2008). Being one of the largest multi-protein complexes in the cell, the NPC consists of multiple copies of ∼30 different proteins known as nucleoporins (Nups), which are organized in an octagonal manner and symmetrically around the cylindrical axis of the NPC (Alber et al., 2007; Tamura et al., 2010; Tamura and Hara-Nishimura, 2013). In addition to components that form the nuclear pore, importins and exportins that carry cargo proteins through the NPC gateway also belong to the NPC family according to the Transporter Classification Database (Saier et al., 2014). A brief overview of the plant NPC components and associated factors is shown in **Figure 1A**. In plants, the NPC family and their associated proteins have been shown to be involved in various biological processes such as responses to auxin, regulation of flowering time, abiotic stress responses, and defense responses to biotic stress (Bond, 2006; Dong et al., 2006b; Parry, 2014). This mini review focuses on the roles of the NPC in plant stress responses, providing an overview of known functions of the NPC and its associated factors in plant responses to abiotic and biotic stress conditions, followed by discussions on the underlying molecular mechanisms.

## Involvements of NPC Components in Plant Stress Responses

The involvements of the NPC family components in plant stress responses were uncovered mostly by isolations of NPC mutants from forward genetic screenings. Diverse mechanisms have been proposed to interpret the dependence on various NPC family components for plant responses to different stress conditions, including cold, abscisic acid (ABA), drought, and biotic stress (Dong et al., 2006b; Verslues et al., 2006; Cheng et al., 2009; Luo et al., 2013). These findings clearly demonstrated the complexity of NPC-mediated plant stress responses. A brief summary of the NPC family components and NPC-associated factors known to be involved in plant stress responses is shown in **Table 1**.

**Table 1:** Nucleoporins and the associated transport factors involved in Arabidopsis stress responses.

Protein	Homologs	Stress	Phenotype	Reference
NUP160 (SAR1)	Human NUP160	Cold	*nup160* mutant is sensitive to chilling stress, and defective in acquired freezing tolerance	Dong et al., 2006b
HOS1	Vertebrate Elys	Cold	Overexpression of HOS1 confers increased sensitivity to freezing stress	Ishitani et al., 1998; Lee et al., 2001; Dong et al., 2006a
LOS4	Yeast DBP5	Cold	*los4-1* is more sensitive to chilling stress; *los4-2* is more tolerant to chilling and freezing stress	Gong et al., 2002, 2005
SAD2	Vertebrate Importin β	ABA, UV-B	*sad2* mutant shows ABA hypersensitivity and more tolerance to UV-B	Verslues et al., 2006; Zhao et al., 2007
KPNB1	Human Importin β1	ABA, drought	*atkpnb1* mutant showed ABA hypersensitivity and enhanced drought tolerance	Luo et al., 2013
MOS3	Human NUP96	Biotic stress	*mos3* mutant exhibited enhanced disease susceptibility to pathogens	Zhang and Li, 2005
MOS6	Yeast Importin α3	Biotic stress	*mos6* mutant exhibited enhanced disease susceptibility to pathogens	Palma et al., 2005
MOS7	Human NUP88	Biotic stress	*mos7* mutant plants exhibit defects in basal and R protein–mediated immunity	Cheng et al., 2009
CPR5	None (a novel transmembrane nucleoporin)	Biotic stress	Loss of function in CPR5 results in resistance against pathogens; while overexpression comprised the resistance	Gu et al., 2016
THP1 (EER5)	Yeast THP1	Ethylene signaling	The mutant showed enhanced ethylene response	Christians et al., 2008
HPR1	Yeast HPR1	Biotic stress and ethylene signaling	Mutation of hpr1 suppress the EDR1 mediated disease resistance and enhance ethylene induced senescence	Pan et al., 2012; Xu et al., 2015

### Cold Stress

In plants, cold stress rapidly induces expression of many transcription factors, including the C-repeat-binding factors (CBFs), which activate transcription of various downstream cold-responsive (*COR*) genes (Chinnusamy et al., 2007; Zhu, 2016). Meanwhile, transcription of *CBF* genes is controlled by their own upstream transcription factors, including the bHLH transcription factor ICE1 (INDUCER OF CBF EXPRESSION 1) (Zhu, 2016). In a genetic screen to search for mutations that impair cold-induced expression of the *CBF3-LUC* reporter gene, AtNUP160 was identified (Dong et al., 2006b). AtNUP160 protein is enriched in the nuclear rim and is critical for nucleo-cytoplasmic transport of mRNAs, as determined by Poly (A)-mRNA *in situ* hybridization (Dong et al., 2006b). The *atnup160-1* mutant plants displayed substantially reduced expression levels of *CBF* genes, which have been shown to be important for acquired freezing tolerance (Gilmour et al., 2000; Lee et al., 2005). Consistent with the reduced induction of *CBF*s, expression of cold-responsive genes were altered in the *atnup160-1* mutant, accompanied by plant phenotypes of being sensitive to chilling stress and being defective in acquired freezing tolerance (Dong et al., 2006b).

In addition to AtNUP160, Arabidopsis HOS1 (high expression of osmotically responsive genes 1) is another clue for NPC involvement in plant stress responses. HOS1 was reported to be physically associated with the NPC components RAE1 (RNA export factor 1) and NUP43, as shown by immunoprecipitation in a proteomic study of plant NPCs (Tamura et al., 2010). Dysfunction of HOS1 resulted in over-accumulation of polyadenylated RNAs in the nucleus (MacGregor et al., 2013). In addition, HOS1 contains a region with homology to the vertebrate nucleoporin Elys (EMBRYONIC LARGE MOLECULE DERIVED FROM YOLK SAC) (Tamura et al., 2010), which is required for recruiting the Nup107–160 complex to chromatin (Gillespie et al., 2007; Doucet et al., 2010). Therefore HOS1 is considered as an NPC component in plants. Interestingly, Arabidopsis HOS1 was initially identified as a negative regulator in cold stress response from a genetic screening using the *RD29A-LUC* reporter system (Ishitani et al., 1997, 1998). In response to low temperature, the *hos1-1* mutation causes enhanced induction of the CBF transcription factors and their downstream cold-responsive genes (Ishitani et al., 1998), while overexpression of HOS1 represses the expression of *CBF*s and their downstream genes as well as confers increased plant sensitivity to freezing stress (Dong et al., 2006a). *HOS1* encodes an E3 ubiquitin ligase which accumulates in the nucleus in response to low temperatures (Lee et al., 2001). Cold stress also induces protein degradation of ICE1 that physically interacts with HOS1 (Dong et al., 2006a). Further investigation showed that HOS1 was required for the ubiquitination and degradation of ICE1 (Dong et al., 2006a). Since ICE1 is a transcription factor that positively regulates cold stress responses in Arabidopsis (Chinnusamy et al., 2003), it was proposed that HOS1 regulates plant cold stress responses through an ubiquitination proteasome pathway (Dong et al., 2006a).

Genetic screening using the *RD29A:LUC* reporter system has also identified LOS4 (low expression of osmotically responsive genes 4), which is essential for mRNA export (Gong et al., 2002). *LOS4* encodes a DEAD-box RNA helicase, which is most closely related to the NPC-associated Dbp5p/Rat8p in yeast (Gong et al., 2002, 2005). The *los4-1* mutant showed reduced gene expression levels of *CBF*s and their downstream targets, as well as increased sensitivity to chilling stress (Gong et al., 2002). Interestingly, the same genetic screening later also isolated another *los4* allele, *los4-2*, which showed enhanced cold induction of *CBF2* and its downstream target genes (Gong et al., 2005). In contrast to *los4-1*, the *los4-2* mutant allele is more tolerant to chilling and freezing stresses, but is sensitive to heat stress (Gong et al., 2005). *In situ* poly(A) hybridization showed that the export of poly(A) RNAs was blocked in the *los4-2* mutant at warm or high temperatures but not at low temperatures, whereas the *los4-1* mutation weakened mRNA export at both low and warm temperatures (Gong et al., 2005), indicating that proper mRNA export is important for plant cold stress responses.

### ABA and Drought Stress

Importin β belongs to a large family of Importin β-like nuclear transport receptors that are also known as karyopherins (Gorlich and Kutay, 1999; Merkle, 2003). Proteins containing the importin β domain can function as either importins that mediate nuclear protein import, or exportins that transport proteins out of the nucleus (Merkle, 2003). The Arabidopsis *SAD2* (*Super sensitive to ABA and drought2*) encodes an importin β-domain family protein and was identified in a genetic screening, which was based on alterations in the expression levels of the stress-responsive *RD29A-LUC* reporter gene (Verslues et al., 2006). The *sad2-1* mutant showed increased luminescence after ABA, salt, cold or polyethylene glycol treatments, and exhibited ABA hypersensitivity in seed germination and seedling growth (Verslues et al., 2006). Although the mechanism underlying ABA hypersensitivity in *sad2* is not clear, SAD2 may function either in importing a negative regulator of ABA response into the nucleus, or in exporting a positive regulator out of the nucleus, given that importin β proteins can function as nuclear transport receptors. Indeed, the ability of SAD2 in transporting proteins was revealed later on. SAD2 was found to be required for nuclear import of MYB4, an R2R3-type transcription repressor that co-immunoprecipitated with SAD2 (Zhao et al., 2007). As a result of the absence of MYB4 protein in the nucleus, *sad2* plants accumulated UV-absorbing pigments and displayed increased tolerance to UV-B radiation (Zhao et al., 2007).

To identify importin genes that function in drought tolerance, Luo et al. (2013) screened T-DNA insertion mutants of Arabidopsis importin β family genes based on the ability to survive after drought treatment. A mutant with a T-DNA insertion in *AtKPNB1* was identified to display considerably increased drought tolerance (Luo et al., 2013). *AtKPNB1* encodes a homolog of human importin β1, inactivation of which resulted in increased stomatal closure in response to ABA, lower rate of water loss, and substantially enhanced drought tolerance (Luo et al., 2013); while over-expression of AtKPNB1 led to increased sensitivity to drought compared to wild type plants, demonstrating that AtKPNB1 is an important negative effector of drought tolerance (Luo et al., 2013).

### Biotic Stress and Ethylene Response

In addition to abiotic stress conditions, biotic stress can also trigger plant responses that involve the NPC. The Arabidopsis *snc1* (*suppressor of npr1-1, constitutive 1*) mutant displays constitutive activation of disease resistance response against pathogens, due to a gain-of-function mutation in a TIR-NBS-LRR-type R gene (Zhang et al., 2003). Genetic screening for suppressors of *snc1* isolated a series of double mutants named *modifier of snc1* (*mos*), which no longer display constitutive resistance to virulent pathogens as the *snc1* single mutant (Palma et al., 2005; Zhang and Li, 2005). Among the *MOS* genes, *MOS3*, *MOS6*, and *MOS7* encode proteins associated with the NPC. *MOS3* encodes a protein with high sequence similarity with human nucleoporin96 (Zhang and Li, 2005). *MOS6* encodes Arabidopsis importin α3 (Palma et al., 2005), while MOS7 is homologous to human and *Drosophila melanogaster* nucleoporin Nup88 (Cheng et al., 2009). Interestingly, it was found that nuclear accumulation of the defense signaling components EDS1 (Enhanced Disease Susceptibility 1) and NPR1 (Non-expresser of PR genes 1) is significantly reduced in *mos7-1* plants (Cheng et al., 2009). Moreover, CPR5 (Constitutive Expresser of Pathogenesis-Related Genes 5), which plays a key inhibitory role in effector-triggered immunity (ETI), was recently found to be a novel transmembrane nucleoporin (Wang et al., 2014; Gu et al., 2016). These findings clearly displayed an important role of nucleo-cytoplasmic trafficking in plant innate immunity.

Nuclear pore complex-dependent plant responses to biotic stress may involve ethylene signaling. In *Nicotiana benthamiana*, Nup75 (Nucleoporin 75) was identified as essential for plant resistance to *Phytophthora infestans*, and for the induction of some defense responses, including ethylene-mediated production of phytoalexin (Ohtsu et al., 2014). Arabidopsis HPR1 (HYPER RECOMBINATION1), which is a component of the NPC-associated TREX (Transcription-Export) complex, was isolated through a screening for *edr1* (enhanced disease resistance) suppressors. In Arabidopsis, *hpr1* mutation not only suppresses the enhanced disease resistance caused by *edr1* mutation, but also increases ethylene-induced senescence in the *edr1* background, suggesting that HPR1 plays a role in ethylene signaling pathway (Pan et al., 2012). It was also found that *hpr1* mutation suppresses plant insensitivity to ethylene as well as *RTE1* (*REVERSION-TO-ETHYLENE SENSITIVITY1*) transcript levels in transgenic Arabidopsis that over-expressed *RTE1* (Xu et al., 2015). An enhanced ethylene response was observed in of Arabidopsis *eer5-1* mutant, which harbors a mutation in the NPC-associated TREX-2 component THP1 (Christians et al., 2008). Enhanced ethylene response in *eer5-1* was correlated with failure to induce appropriately a subset of ethylene-regulated genes (Christians et al., 2008). Therefore, the NPC and its associated RNA export complexes may be involved in ethylene-mediated plant stress responses.

## Molecular Mechanisms of NPC-Dependent Plant Stress Responses

In eukaryotes, mRNAs synthesized in the nucleus need to be exported to the nucleoplasm for protein production; whereas nuclear proteins such as transcription factors must be imported, after protein synthesis in the cytoplasm, into the nucleus for proper function. Thus, roles of the NPC in plant stress responses are often attributed to NPC’s function in controlling RNA/protein trafficking between the nucleus and the cytoplasm. In addition, it has also been shown that the NPC and its associated factors can be involved in certain biological processes through gene regulation at the transcriptional level.

### Selective mRNA Export

By using *in situ* hybridization, many studies of nucleoporin mutants have demonstrated accumulation of polyadenylated mRNA in the nucleus (Dong et al., 2006b; Lu et al., 2010; Parry, 2014). Interestingly, stress conditions can also induce bulk mRNA accumulation in the nucleus (Saavedra et al., 1996; Bond, 2006; Muthuswamy and Meier, 2011). It is thus important to understand how certain mRNAs are selectively transported under stress conditions. In yeast, following heat or ethanol stress, poly(A) RNAs accumulates within nuclei, while mRNAs encoding Hsps (heat shock proteins) are efficiently exported from the nucleus (Saavedra et al., 1996; Bond, 2006). Recently, it was revealed that in yeast, cellular stress induces dissociation between regular mRNAs and the export receptor Mex67 as well as its adaptor proteins, thereby preventing general mRNA export; meanwhile, heat-shock mRNAs are efficiently exported in association with Mex67, without the need of adapter proteins (Zander et al., 2016). In fact, adaptor-bound mRNAs, but not free mRNAs, undergo quality control, indicating that at the cost of accuracy, heat-shock mRNAs are exported and translated without delay, allowing cells to survive extreme situations (Zander et al., 2016).

In Arabidopsis under heat or ethanol stress, a correlation seems to exist between altered protein sumoylation levels and bulk mRNA nuclear retention (Muthuswamy and Meier, 2011). Exposing Arabidopsis plants to heat shock and ethanol stress both resulted in elevation in high-molecular-weight SUMO conjugates, accompanied by nuclear mRNA accumulation (Muthuswamy and Meier, 2011). Because mutations in either SUMO E3 ligase SIZ1 or SUMO isopeptidase ESD4 (Early in short days 4) leads to nuclear mRNA retention, it has been suggested that sumoylation acts upstream of mRNA export, likely through the transient sumoylation status of one or more factors involved in mRNA trafficking (Muthuswamy and Meier, 2011).

### Control of Protein Transport

Trafficking from the cytoplasm to the nucleus is essential for proteins with nucleus-specific functions. Under stress conditions, nuclear import of certain proteins can be critical for the plant to reprogram cellular processes to combat the stress. In Arabidopsis, MYB4 negatively regulate the transcription of cinnamate 4-hydroxylase (C4H) and thereby synthesis of sinapate esters which are UV-absorbing pigments (Zhao et al., 2007). MYB4 was found to co-immunoprecipitate with SAD2, which is an importin β-domain family protein essential for nuclear import of MYB4 (Zhao et al., 2007). Consistently, Arabidopsis *sad2* mutant is more tolerant to UV-B radiation compared with wild type plants. Protein interaction between MYB4 and SAD2 requires the conserved GY/FDFLGL motif in the C terminus of MYB4, as demonstrated by the observation that an Asp to Asn mutation in the GY/FDFLGL motif abolishes the interaction between MYB4 and SAD2 (Zhou et al., 2015). Without a functional GY/FDFLGL motif, MYB4 failed to be transported into the nucleus and thus cannot repress their target genes (Zhou et al., 2015).

Besides SAD2, Arabidopsis MOS7 is another plant nucleoporin that has been shown to regulate nuclear accumulation of stress-responsive proteins. MOS7 is homologous to human and Drosophila nucleoporin Nup88 (Cheng et al., 2009). In animals, Nup88 attenuates NES (nuclear export signal)-mediated protein nuclear export (Roth et al., 2003; Xylourgidis et al., 2006). In Arabidopsis, the *mos7-1* mutation caused defects in basal and R protein–mediated immunity and in systemic acquired resistance (Cheng et al., 2009). Further investigation showed that nuclear accumulation of the autoactivated R protein snc1 as well as the defense signaling components EDS1 (Enhanced Disease Susceptibility 1) and NPR1 (Non-expresser of PR genes 1) was significantly reduced in *mos7-1* plants, whereas nuclear abundance of other tested proteins was unaffected (Cheng et al., 2009), supporting the notion that trafficking of certain stress-responsive proteins can be subject to selective regulation by nucleoporins.

Despite the obvious specificity of the connection between the NPC and plant stress responses, little is known about how such specificity is achieved. Arabidopsis CPR5 (Constitutive Expresser of Pathogenesis-Related Genes 5) was initially identified as a negative regulator of plant Programmed Cell Death (PCD) and Effector-Triggered Immunity (ETI) (Boch et al., 1998; Wang et al., 2014). Loss-of-function mutations in CPR5 resulted in resistance against multiple pathogens, whereas over-expression of CPR5 compromised ETI-associated PCD and pathogen resistance in Arabidopsis (Boch et al., 1998; Wang et al., 2014). CPR5 was later revealed as a transmembrane nucleoporin (Wang et al., 2014; Gu et al., 2016). In addition, transient interference of CPR5 expression yielded in transcriptome patterns that significantly match plant responses to a variety of stress conditions including cold, salt/osmotic stress, abscisic acid, and various pathogens (Gu et al., 2016). It was hypothesized that with compromised CPR5 function, the NPC adopts a structure with significantly increased permeability and/or transport activity that allows deregulated nuclear influx of diverse signaling cargos, which normally undergo nuclear translocation only under stimulus-induced conditions (Gu et al., 2016). Indeed, over-expression of CPR5 caused substantial cytoplasmic retention of NPR1, JAZ1, and ABI5, which are stress- and phytohormone-related nuclear proteins (Wang et al., 2014; Gu et al., 2016). Researchers further revealed that upon activation by immunoreceptors, CPR5 undergoes an oligomer-to-monomer conformational switch, which reconfigures the selective barrier to allow significant influx of nuclear signaling cargos through the NPC (Gu et al., 2016). These findings thus established CPR5 as a converging point in the specific connection between the NPC and the ETI/PCD responses in plants.

### Regulation of Gene Expression

In addition to regulating RNA/protein transport, the NPC can also regulate gene expression at the transcriptional level. In as early as 1985, the “gene gating” hypothesis has proposed that certain expanded chromatin regions with transcription units can be attached to the NPC (Blobel, 1985). Subsequently this hypothesis has been supported by many studies in yeast and Drosophila. In yeast, several highly inducible genes are randomly distributed in the nucleoplasm when transcriptionally repressed but are recruited to the nuclear periphery upon activation (Brickner and Walter, 2004; Casolari et al., 2004; Taddei et al., 2006). In Drosophila, the nucleoporins Sec13, Nup98, and a subset of FG-repeat nucleoporins bind to developmentally regulated genes that are undergoing transcription induction (Capelson et al., 2010). Interestingly, certain NPC target genes exhibit transcriptional memory, i.e., after being repressed, these genes remain at the nuclear periphery for several generations and are primed for reactivation (Light et al., 2010). Transcriptional memory of yeast *INO1* requires the NPC component Nup100, as well as incorporation of the histone variant H2A.Z after gene repression (Light et al., 2010), indicating that the NPC can directly interact with chromatin and contribute to epigenetic gene regulation in response to developmental and environmental stimuli.

In plants, limited information is available for the mechanism of how the NPC regulates gene transcription. Arabidopsis *hos1* mutant exhibits an early flowering phenotype with repressed *FLC* gene expression. In an effort to explore how HOS1 regulates *FLC* (*FLOWERING LOCUS C*) expression, researchers (Jung et al., 2013) found that HOS1 binds to *FLC* chromatin in an FVE-dependent manner. In addition, HOS1 binding to the *FLC* locus is dramatically elevated at 4°C. HOS1 also interacts with the histone deacylase HDA6 and inhibits the binding of HDA6 to the *FLC* locus. Therefore, it was proposed that under short-term cold stress, HOS1 binds to *FLC* chromatin in an FVE-dependent manner to limit the chromatin accessibility to HDA6, allowing for activation of *FLC* transcription (Jung et al., 2013). Recently, our work isolated Arabidopsis SAC3B (SUPPRESSOR OF ACTIN3B), a core component of the TREX-2 (transcription-coupled export 2) complex, through a forward genetic screening for anti-silencing factors (Yang et al., 2017). Mutation of SAC3B caused gene silencing of a reporter gene luciferase driven by double 35S (d35S) promoter, accompanied by elevation in the repressive histone mark H3K9me2 and by reduction in RNA polymerase Pol II occupancy (Yang et al., 2017). Moreover, THP1 (Tho2/Hpr1 PHENOTYPE1) and NUA (NUCLEAR PORE ANCHOR) were identified as SAC3B-associated proteins whose mutations also caused d35S::LUC silencing (Yang et al., 2017). The THP1 is another representative component of TREX-2 complex, while NUA is homologous to a component of nuclear pore basket in vertebrate (Krull et al., 2004; Jacob et al., 2007). Importantly, *FLC* gene expression is decreased in both *sac3b* and *nua* mutants (Xu et al., 2007; Yang et al., 2017), while our unpublished IP-MS results also identified HOS1 as a SAC3B-interacting protein. Thus it appears that the Arabidopsis NPC, in association with the TREX-2 complex, controls gene expression through HOS1-dependent chromatin targeting. In the future, it would be interesting to examine whether stress-inducible genes are tethered to the NPC, and whether stress memory can be mediated through chromatin tethering to the NPC.

## Concluding Remarks

It has been clear that a functional NPC is important to plants under stress conditions, and that involvements of the NPC family and the associated factors in plant stress responses can be mediated through different mechanisms including control of mRNA/protein trafficking and transcriptional gene regulation (**Figure 1B**). However, insights into the underlying mechanisms are still largely unclear. A key focus would be the target specificity of the NPC under stress conditions. It would also be important to understand how NPC components perceive stress signals, as well as to fully depict the involvement of NPC-dependent transcriptional regulation in plant stress responses.

## Author Contributions

YY and HZ drafted the manuscript. YY, WW, ZC, J-KZ, and HZ revised and finalized the manuscript.

## Conflict of Interest Statement

The authors declare that the research was conducted in the absence of any commercial or financial relationships that could be construed as a potential conflict of interest.
